# miRNAomic Approach to Plant Nitrogen Starvation

**DOI:** 10.1155/2021/8560323

**Published:** 2021-11-09

**Authors:** Peerzada Yasir Yousuf, Peerzada Arshid Shabir, Khalid Rehman Hakeem

**Affiliations:** ^1^Department of Botany, Government Degree College Pulwama, 192301, Jammu and Kashmir, India; ^2^Department of Botany, Government Degree College Sopore, 193201, Baramulla, Jammu and Kashmir, India; ^3^Department of Biological Sciences, Faculty of Science, King Abdulaziz University, Jeddah, 21589, Saudi Arabia; ^4^Princess Dr. Najla Bint Saud Al-Saud Center for Excellence Research in Biotechnology, King Abdulaziz University, Jeddah, Saudi Arabia

## Abstract

Nitrogen (N) is one of the indispensable nutrients required by plants for their growth, development, and survival. Being a limited nutrient, it is mostly supplied exogenously to the plants, to maintain quality and productivity. The increased use of N fertilizers is associated with high-cost inputs and negative environmental consequences, which necessitates the development of nitrogen-use-efficient plants for sustainable agriculture. Understanding the regulatory mechanisms underlying N metabolism in plants under low N is one of the prerequisites for the development of nitrogen-use-efficient plants. One of the important and recently discovered groups of regulatory molecules acting at the posttranscriptional and translational levels are microRNAs (miRNAs). miRNAs are known to play critical roles in the regulation of gene expression in plants under different stress conditions including N stress. Several classes of miRNAs associated with N metabolism have been identified so far. These nitrogen-responsive miRNAs may provide a platform for a better understanding of the regulation of N metabolism and pave a way for the development of genotypes for better N utilization. The current review presents a brief outline of miRNAs and their regulatory role in N metabolism.

## 1. Introduction

The expanding global population and increased food demands call for enhanced plant production, which in turn depends primarily on the nutrition status of the plants. Plants need different mineral nutrients for their proper growth and development. N is one of the most essential macronutrients required by plants for their optimal growth, development, productivity, reproduction, fitness, and survival [1]. It is involved in various physiological and metabolic processes including amino acid metabolism, carbon assimilation, and protein synthesis. N forms a key constituent of different plant biomolecules including proteins, nucleic acids, chlorophylls, enzymes, energy storage, and transfer compounds, like ATP (adenosine triphosphate), signaling molecules, secondary metabolites, quaternary ammonium compounds, plant hormones, and polyamines [2, 3]. Owing to its role and importance in plants, it may not be wrong to state that N represents plant life. As far as the quantity of mineral nutrients needed by the plants is concerned, N comes first in the list. Despite its importance, N is the key limiting nutrient in agriculture. Low concentrations of N limits plant growth, development, and productivity. To acquire maximum yields for the burgeoning population, there has been an increased use of synthetic N fertilizers. The current global annual N fertilizer demand is estimated to be more than 108 million tonnes [4]. However, there are some negative aspects associated with this dramatically increased utilization of N fertilizers. First is the high cost of N fertilizers. The use of N fertilizer is classically the single principal input cost for numerous crops, and since its production is energy-intensive, this cost is reliant on the price of energy [5]. Secondly, most of the N added to the soil is not utilized by the plants and is lost to the environment. Usually, only less than half of the applied N is taken up by the plant depending on the species and cultivar. The remaining larger proportion of N gets subjected to surface run-off, leaching of nitrates, ammonia (NH_3_), volatilization, or bacterial competition finally finding its way to the environment [6]. The negative impacts of N on the environment are becoming all the time more obvious [7, 8]. The accumulation of the excess nitrates in freshwater bodies leads to eutrophication resulting in algal blooms which in turn produce a hypoxic environment resulting in the significant loss of aquatic life forms [9]. The production process and excessive usage of N fertilizers have also been implicated to play crucial roles in both global warming and stratospheric ozone depletion [10]. For example, nitrous oxide (N_2_O), the third most abundant greenhouse gas (GHG), after only carbon dioxide and methane [11], is the product of both anthropogenic and natural processes [11] and is 300 times more potent GHG than CO_2_ [12]. Although the emission of this GHG is largely attributed to microbial nitrification and denitrification (both natural processes), however, the addition of N fertilizers to soil accelerates the production of nitrous oxide, giving this process an anthropogenic effect. Economically, N pollution is projected to cost the global economy 200-2000 billion USD annually, which corresponds to 0.2%-2% of global GDP [13].

In this context, to solve this conundrum and to allow sustainable, eco-friendly food production, there is a great need to develop plants/or genotypes that are less dependent on the injudicious N overfertilization. This can be achieved by increasing the N uptake efficiency and the utilization efficiency of the absorbed N. It has been estimated that an increase of only 1% of N use efficiency may save 1.1 billion dollars annually [14].

Nitrogen-use-efficiency (NUE) in plants is a complex trait that relies both on the availability of N in the soil and the mode of usage of N. While the former factor is affected by various environmental factors including temperature, precipitation, soil type, soil salinity, wind, and pH, the latter depends on several important processes including uptake, assimilation, translocation, and recycling and remobilization (during senescence) [15, 16].

Understanding the mechanisms underlying plant adaptation to N deficiency is imperative for improving NUE and trimming down chemical N fertilization. To sense the nutrient deficiency and to turn on adaptive mechanisms, plants have evolved various mechanisms at physiological, biochemical, and molecular levels [17]. At the molecular level, plants trigger a network of genes together with their altered expression via transcriptional, and/or translational regulations. Some of the genes are upregulated (mainly the protective ones) while some are downregulated (mainly the negative regulators). One of the intricate molecular mechanisms operative at the post-transcriptional and/or translational level to regulate the genes adapted to the N-deficient environments is the small regulatory microRNA.

## 2. MicroRNAs and Their Mode of Action

MicroRNAs (miRNAs) are an important class of small, endogenous, noncoding, single-stranded, regulatory RNA molecules almost 21-24 nucleotides long and are produced from the precursors with intramolecular stem-loop structures through endonucleolytic processing [18]. The miRNAs are extensively distributed in biological organisms and are highly conserved (evolutionary) in plants [19, 20]. Based on the sequence complementarity miRNAs bind to specific sites in the 3′ UTR region of the target mRNA and facilitate posttranscriptional silencing through degradation or/and translational inhibition of mRNA [19–21].

**Figure 1:**
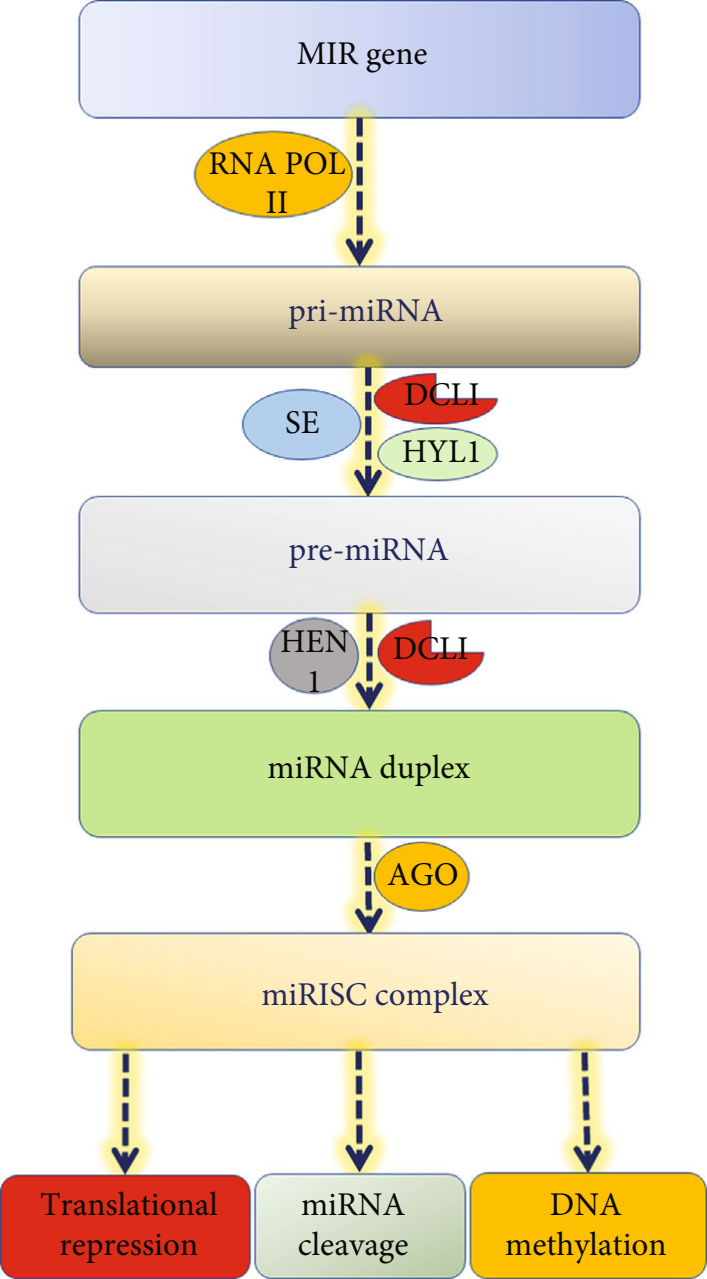
Schematic diagram depicting the biogenesis and mode of action of plant microRNAs.

The miRNA regulates the expression of their target genes in three main ways (Figure 1) as follows:

- Cleavage of target mRNA: One of the main mechanisms of the negative gene regulation by miRNA is the degradation of the target mRNA. The miRNAs based on their complementarity sequences pair with the target mRNA and result in the cleavage of the target mRNA within the region of pairing. This process of cleavage which occurs on RER [22] and yields 5′ and 3′ cleavage products is catalyzed by the PIWI domain of AGO proteins. The PIWI domain of AGO proteins forms a fold (analogous to RNase H) and constitutes the main catalytic center [23–26]. Different AGO proteins may possess this catalytic activity, e.g., in *Arabidopsis*, AGO1, AGO2, AGO4, AGO7, and AGO10 are known to exhibit this catalytic action [27–31]. The miRISC uncapped and sensitive 3′ cleavage products are degraded by the exonucleases (EXONUCLEASE4 being identified while the other nucleases were still unknown). On the other hand, capped 5′ cleavage products first undergo uridylation by HESO1 [32] and are then cleaved by RISC-INTERACTING CLEARING 3′-5′ EXORIBONULEASE 1 (RICE1) and (RICE2) [33] assisted by some RNA exosome cofactors including SKI2, SKI3, and SKI8 [34]
- Repression at the translational level: In addition to target messenger RNA cleavage, miRNAs negatively regulate the gene expression through translational repression also [35]. In the case of plants, although miRNAs and their target mRNAs have mostly near-perfect complementarity in their sequences, nevertheless, the negative regulation via translational repression is extensive [36]. This process is facilitated by DRB2 which represses the expression of HYL1 [37]. The translational repression is carried out by many AGO-miRISC complexes including AGO1-miRISC, AGO7-miRISC, and AGO10-miRISCs [36, 38] through different mechanisms, e.g., AGO1-miRISCs execute the repression (1) by targeting 5′ untranslated regions and blocking ribosome recruitment and translation initiation or (2) by targeting open reading frames (ORFs) and blocking ribosome movement and translation elongation [39]. The execution of this repression is assisted by multiple factors including KATANIN [36], VARICOSE [36], SUO [40], and ALTERED MERISTEM PROGRAM1 (AMP1) [41]
- DNA methylation: Although miRNAs act mainly at the post-transcriptional level, yet some studies suggest their role at the transcriptional level by methylating DNA. For example, one of the prerequisites required for the methylation of PHB and PHV genes is the complementarity between PHB and PHV mRNAs and miR165/166 [42]. Another example is the directed methylation of target loci-derived transcripts in rice by effector AGO4 which comprises long miRNAs processed from pri-miRNAs by DCL3 [43]

## 3. Biological Functions of MicroRNAs in N Metabolism

The microRNAs are known to regulate various processes associated with plant growth and development [22, 44–46]. Besides, there are several miRNA families which are expressed during the different stress conditions including nutrient stress and modulate the adaptive responses [47–49]. With the advancement in molecular techniques including deep sequencing technology, a large number of plant miRNA families with important roles in mediating plant tolerance to N stress have been identified and characterized. These small regulatory molecules are implicated in different N metabolic processes including uptake, transport, and assimilation and regulate the use of N and partake in the plant adaptation to N deficiency [50].

Numerous studies have demonstrated the changes in the expression level of different miRNA families in N limitation conditions in various plant species such as *Arabidopsis* [51, 52], maize [53, 54], sorghum [55], rice [32], and soybean [33]. The altered expression pattern of these miRNAs has been shown to play crucial roles in plant adaptive responses to N limitation via the mediation of the expression of their target genes involved in N uptake and remobilization [56]. Although a number of miRNAs responsive to N stresses have been identified via changes in their expression profile [51, 57, 58], few of them have been characterized.

The comparison of the identity and expression profile of different miRNAs responsive to N stresses reveals several common features among these key regulators. In the majority of the cases, it has been observed that some miRNAs are upregulated, while others are downregulated, under N limiting conditions, depending on the species, tissues, and experiment design. For instance, in *Arabidopsis*, upregulated miRNAs include miR156, miR160, and miR447 while miRNAs including miR169, miR397, miR398, miR399, miR408, and miR827 were significantly downregulated under N starvation. A study by Li et al. [59] demonstrated the altered expression of twenty miRNAs including miR160, miR167, miR393, miR396 etc. under N deficient conditions in peanut. Analysis of miRNA expression in root tissues of several rice genotypes showed that miR156 and miR447 were overexpressed while miR398 was downregulated in response to N stress [32]. The different miRNAs which get differentially expressed under N starvation conditions are given in Table 1.

## 4. miRNAs and Their Targets under Low N

Since miRNAs selectively regulate the expression of their target genes under specific conditions, so it is crucial to identify and analyze these target genes. Using computational prediction tools, we can easily identify these targets as plant miRNAs normally recognize their target genes through complementary base-pairing. Some data indicate that the miRNAs target multiple targets belonging mostly to plant transcription families and proteins/enzymes involved in various metabolic pathways including various stress responses [60, 61].

### 4.1. miR156

In *Arabidopsis*, it has been demonstrated that miR156 regulates the SPL (Squamosa Promoter Binding Protein Like) gene family and targets 11 genes out of its 17 members [62–65]. This SPL gene family regulates leaf formation, anthocyanin biosynthesis, vegetative phase change, flowering, and fertility [66–68]. Transgenic plants of *Arabidopsis*, rice, tomato, and maize with upregulated expression of miR156 resulted in prolonged phase transitions and stunted growth [69–72]. Emerging data from the analysis of the transcriptomics of N-limited plants, it has been demonstrated that one of the miR156/SPL3 modules might act by repressing the vegetative phase change under limiting the availability of N [73]. Overexpression of miR156 also promotes increased anthocyanin biosynthesis (through SPL9) [74] causing redness and yellowing of leaves, a key feature of the plant N starvation response [75].

### 4.2. miR160

Under N starvation, the overexpressed miR160 has been observed to enhance the production of lateral roots in *Arabidopsis* through the downstream effect on members of the auxin response factor (ARF) family [51]. Mallory et al. demonstrated that ARF10, ARF16, and ARF17 mRNAs are the specific targets of miR160 in *Arabidopsis* [76]. Through more advanced techniques, studies showed that the overexpressed miR160 exerts its regulatory effect on the root gravity sensing and the number of lateral roots produced through the ARF10- and ARF16-mediated downstream pathways [77]. Likewise, fewer lateral and adventitious roots were observed in *Arabidopsis* plants that demonstrated increased levels of ARF17 mRNA due to impaired miR160 regulation [78]. Studies showed that transgenic plants with upregulated levels of miR160 had developed more lateral and adventitious roots implying that under N deficiency, the induced overexpression of miR160 might promote lateral and adventitious root growth via the mediation of ARF16 and ARF17 [52]. These pieces of information suggest that miR160 upregulation under N starvation conditions may have a role in promoting lateral root growth to access additional N.

### 4.3. miR164/miR167

miR164 is reported to target and cleave the gene transcripts containing the NAC domain, including NAC1, which are involved in auxin-induced signal transduction for the development and growth of lateral roots [79]. Under low nitrate conditions, miR164 is upregulated, while the transcript levels of *NAC1* genes are downregulated [80]. Plants with downregulated expression of miR164 concomitant with the upregulation of gene transcripts containing the NAC domain show more lateral root growth [79]. Under the N-limiting conditions, miR167 in *Arabidopsis* is reported to target two Auxin Response Factors ARF6 and ARF8 and mediates the plant growth and development of lateral roots [81]. For instance, the expression levels of ARF8 are upregulated in pericycle and lateral root cap cells under N-deficient conditions and regulate the growth and development of lateral roots [82].

ARF proteins induce or suppress the expression of the gene in response to the plant phytohormone auxin by binding to auxin-responsive *cis*-acting promoter elements [83, 84]. Cell-specific profiling has revealed that in pericycle cells, nitrate or glutamine/glutamate downregulates the expression of miR167, permitting the target ARF8 transcript to accumulate and trigger lateral root formation in *Arabidopsis* [82]. miR167 has also been observed to target IAA-Ala resistant-3 (IAR-3), whose protein releases bioactive auxin by hydrolyzing the inactive auxin derivative indole-3-acetic acid alanine, for the growth and development of lateral roots under high osmotic stress [85]. Therefore, the downregulated miR167 expression under N-limiting condition might lift its inhibitory effect on auxin transcription factors which in turn could induce the development of lateral roots [52]. Collectively, these studies suggest that the availability of N alters the miRNA expression levels that in turn regulate the auxin signaling by targeting ARF8 and IAR3 which subsequently regulates the architecture of the root system.

### 4.4. miR169

miR169 is a large miRNA family and targets family members of NUCLEAR FACTORY Y, SUBUNIT A (NFYA), coding for nitrate transporter (NRT1.1 and NRT2.1.) in *Arabidopsis*. Under N deficiency, the expression of miR169 gets downregulated while the transcript levels of NFYA genes are upregulated [51, 86]. Moreover, transgenic overexpression of miR169 represses the NFYA expression, causing *Arabidopsis* plants to accumulate less N, and was more sensitive to N deficiency as compared with wild-type plants showing lower chlorophyll content, higher anthocyanin concentration, and early senescence phenotype. This hypersensitivity in transgenic miR169-overexpressing plants to N starvation has been attributed to the N-uptake capacity since NFYA regulates the nitrate transporters NRT1 and NRT2 [86] suggesting an important role of miR169 in uptake and remobilization [52].

### 4.5. miR393

miR393 and one of its targets AFB3 (auxin-signaling F-box protein 3) comprise a unique N-responsive regulatory module, implicated in controlling the growth and development of lateral root system in response to the external and internal N availability [82, 87]. The availability of nitrate in tips and pericycle regions of roots upregulates the transcription of AFB3, thereby inhibiting the growth of primary root and induction of lateral root growth.

However, the N metabolites produced from the reduction and assimilation of nitrate upregulate miR393 expression, specifically causing the downregulation of AFB3, which in turn regulates the growth of primary and lateral roots [87]. This upregulation of miR393 by N metabolites and the corresponding rapid downregulation of its target AFB3 by miR393 constitutes a finely regulated feedback module that allows the plants to precisely adjust their root system architecture depending on external and internal nitrate availability.

### 4.6. miR397/408

miR397 has been reported to target members of the family laccase (LAC2, LAC4, and LAC17), the copper-containing enzymes regulating the diverse range of functions related to defense mechanism and lignification of the cell wall [88, 89]. miR397 and miR398 are involved in both N and Cu homeostasis; however, under N stress, they get downregulated, while in response to Cu deficiency, they get upregulated [19]. Using massive sequencing and computational analyses, Liang et al. [52] demonstrated that the laccase family is also the potential target for miR408 and miR857 which get downregulated in *Arabidopsis* under N-limiting conditions. Abdel-Ghany and Pilon [21] demonstrated that these miRNAs target the genes of family laccase, coding for the copper-containing enzymes, and are involved in a diverse range of functions (miR408: LAC3, LAC12, and LAC13; miR857: LAC7). As far as the regulation mechanism is concerned, it has been predicted that miR397, miR408, and miR857 regulate the activity of the laccase family by maintaining the C : N homeostasis. Metabolic profiling has revealed an increase in carbohydrate and starch levels under N-limiting conditions [90, 91], and the excess fixed C gets incorporated into lignin through increased laccase activity.

### 4.7. miR399

miR399 has been described in the literature as a key regulator in phosphate metabolism, which gets upregulated in phosphate-limiting conditions, targeting the PHO_2_ gene encoding for ubiquitin-conjugating E2 enzyme UBC24 [92], a membrane-associated putative Pi transporter, responsible for the degradation of PHO_1_ [93]. During phosphate starvation, PHO_2_ transcripts are cleaved by miR399, releasing the posttranslational repression of PHO_1_, thus allowing this protein to accumulate and participate in phosphate uptake. Conversely, the downregulated expression of miR399 presumably permits higher-level expression of UBC24, thereby enhancing proteasome-mediated N remobilization of other unidentified targets such as Rubisco. Alternatively, the upregulation of miR399 and the resulting decreased phosphate transport could represent an additional mechanism that conserves plant resources in the form of high-energy phosphate compounds. The intimate cross-talk between N response and P pathways at the level of regulated proteolysis is not well characterized; however, some data indicate that the N : P link is regulated by PHO_2_ and miR399 [94].

### 4.8. miR444

The monocot-specific miR444 is specifically upregulated by N deficiency in bread wheat [95]. Li et al. [96] carried out the expression analysis of transgenic plants of rice and demonstrated an upregulation of four MIKC-type MADS-box transcriptional factor genes (OsMADS23, OsMADS27a, OsMADS27b, and OsMADS57). Phylogenetic analysis grouped these MADS-box transcription factor targets of miR444 with *Arabidopsis* ANR1 clade [97], which is an important regulator in the NO_3_ signaling pathway and thus mediating plant tolerance to the N starvation stress via lateral root growth [98].

### 4.9. miR528

miR528 is another monocot-specific miRNA that has the potential to regulate multiple stress responses. In maize, miR528 is downregulated in response to low N availability, releasing the posttranslational repression of its targets, with miR528 transgenic plants exhibiting increased transcript levels of NiR which might contribute to better NUE and the increased efficiency for N assimilation [99]. Additionally, in another study, Ascorbic Acid Oxidase (AAO) and Copper Ion Binding Protein 1 (CBP1) have been reported to be two putative targets of miR528 and are significantly repressed in miR528-overexpressing transgenic plants [99]. Expression and functional studies have revealed that both the targets mediate oxidation homeostasis and, thus, prevent damage to cellular components. Transgenic plants expressing lower levels of AAO under N deprivation maintain relatively high levels of redox AA, thereby keeping the balance between reactive oxygen species (ROS) production and its scavenging under oxidative stress.

### 4.10. miR827

miR827 is another miRNA that attracted our attention. The known target genes of miR827 are members of SYG1/PHO81/XPR1(SPX) domain-containing proteins. Under N-limiting conditions, miR827 represses the expression of the SPX domain-containing N limitation adaptation (NLA) gene, which is an essential component for developing the N limitation adaptation responses [100]. Disruption of NLA in *Arabidopsis*, under limiting N conditions, has also been linked to reduced photosynthetic capacity, increased biosynthesis of anthocyanin, and caused plants to undergo early senescence. Both anthocyanin biosynthesis and N remobilization are key features of tolerance to N starvation.

Apart from the above-mentioned miRNAs that are differentially expressed under N starvation where nitrate acts as the source, there is also an effect of the type of N source. Li et al. [101] worked on the identification of 11 miRNAs in rice roots that are differentially expressed in response to ammonium. These included OsmiR6250, OsmiR5082, OsmiR1846d-5p, OsmiR319a-3p, OsmiR159a.1, OsmiR529a, OsmiR818b/e, OsmiR394, OsmiR159f, OsmiR167h-3p, and OsmiR818.

## 5. Role of MicroRNAs in the Legume-*Rhizobium* Symbiosis

Besides their well-studied functions in N sensing and signaling, miRNAs are also being characterized for their role in plant-microbe symbiosis. To date, large numbers of symbiosis-responsive miRNA families have been identified to regulate different stages of nodule development [102], including several highly upregulated miRNAs in mature nodules [103, 104]. In this context, an earlier study conducted by Subramanian et al. [105] predicted and identified several miRNAs as participants in developing this symbiotic response. The massive sequencing and computational analysis of miRNA library derived from whole soybean roots inoculated with *Bradyrhizobium japonicum* revealed that some miRNAs, like miR168 and miR172, were firstly upregulated immediately one hour postinoculation, while a decreased pattern of expression was observed after this point. On the other hand, the expression levels of miR160 and miR169 were downregulated in response to the inoculation of rhizobial bacteria, suggesting the dynamic role of specific miRNAs in modulating signaling and nutrient homeostasis during inoculation. Previously, Wang et al. [66] constructed sRNA libraries from soybean root tips and found that five miRNA families in soybean (miR172, miR396, miR1508, miR1509, and miR2107) are crucial for the establishment of legume-*rhizobium* symbiosis. *In situ* analysis of selected miRNAs by authors demonstrated that novel miRNAs miR172 and miR2107 accumulated in root nodules while miR396, miR1508, and miR1509 were downregulated in functional nodules during symbiotic N fixation.

Turner et al. [102] reported that the downregulated expression of miR160 resulted in a significant decrease in the nodulation process, while the number of mature nodule formation was significantly increased upon the elevated expression of miR482, miR1512, and miR1515 compared with transgenic control vector roots upon *B. japonicum* inoculation [106]. Barros-Carvalho et al. [107] demonstrated the differential expression of miR1530, miR1520, and miR1522 during the early stages of nodule development, facilitating nodule organogenesis and symbiotic interactions. In breakthrough with soybean roots, Wang et al. [108] proved that the miR172 modulates both *Rhizobium* infection and nodule organogenesis. Ectopic expression of miR172 caused a dramatic increase in nodule initiation and mature nodule numbers. Yan et al. [109] in their experimentation with soybean roots explored four miRNAs (Gma-miR2606b, miR1514, TAG2382310, and Gma-miR4416) for their functional relevance with plant-rhizobial symbiosis and demonstrated that the transcripts of miR1514 and TAG23822310 had no significant effect on nodulation while upon the upon rhizobial treatment, the expression levels of both GMa-miR2606b and Gma-miR4416 were downregulated. However, in transgenic roots, the upregulation of Gma-miR2606b led to a significant increase in several mature nodules, while the number of nodule formations was drastically reduced upon constitutive upregulation of Gma-miR4416 [109].

The potential targets of these symbiosis-responsive miRNAs mostly belong to families of various transcription factors (TFs) and proteins, related to various metabolic pathways or stress responses including hormone-mediated signaling, developmental-related proteins, and defense-associated responses as well as nitrate transporters. De Luis et al. [110] demonstrated that in *L. japonicas*, the miR171c targets the transcription factor GRAS which acts downstream of CCaMK and Ca^2+^ spiking in the Nod factor signaling pathway [111]. In soybean, miR171o and miR171q also target the GRAS family of transcription factors [112, 113]. Similarly, miR396 shows differential expression patterns in roots during the nodule formation and growth of lateral roots [114]. The authors demonstrated that the miR396 limits symbiotic colonization by regulating the growth-factor-regulating gene (GRF) [114]. In infected root hairs and during the early stages of nodule primordia formation, miR172a promoter is induced [115] through miR172 silencing of the APETALA2 (AP2) transcription factor [115, 116]. The targeted AP2 transcription factor in the case of soybean has been reported to be Nodule Number Control 1 (NNC1) which binds directly to the promoter of the early nodulin gene ENOD40 and thus regulates the nodule primordium formation [108].

miR166 has been reported to target a conserved class- III homeodomain-leucine zipper (HD-ZIPIII) transcription factor family [117], and the ectopic upregulation of miR166 negatively regulates the density of lateral roots, nodule number, and vascular differentiation in both roots and nodules in *M. truncatula* [118]. Similarly, miR169 has been reported to regulate meristem maintenance and bacterial release in nodules by controlling the spatial distribution of the transcription factor HAP2-1 [117]. While both miR166 [118] and miR169 [117] are thus likely to be involved in nodule organogenesis, no miRNA has so far been linked to N fixation activity in nodules.

## 6. Conclusion

miRNAs are currently emerging as the most interesting gene regulators in plants during mineral nutrient stress. In the past few years, significant progress has been made to analyze and characterize different N-responsive miRNAs, with an increasing number of research reports on the dynamic roles of miRNAs while maintaining an optimal supply of N under stress situations. With the advent of new molecular techniques, several key N-responsive miRNAs have been identified, which has opened a parallel avenue of research in deciphering their regulatory roles, resulting in the potential to find target genes that were previously not known for their involvement in nutrient response. Moreover, while controlling the expression of its target genes, miRNAs seem to act on transcription factors, which is essential for wide-scale dynamic regulation to efficiently control nutrient homeostasis across different cells/tissues within a plant. Therefore, microRNA-based manipulation of miRNA expression levels would represent an effective strategy to overexpression of mRNAs for engineering NUE in plants, particularly in light of recent studies on miRNA expression patterns in response to N starvation.

## Figures and Tables

**Table 1:** Differentially expressed miRNAs under nitrogen starvation condition.

miRNA	Target/function	Mode of regulation	References
miR156	Squamosa promoter binding protein-like (SPL) transcription factors	Up	[119–128]
miR159	MYB, TCP transcription factors/peptidase C48, SUMO/sentrin/Ubl1	Up	[119, 124–126, 129–134]
miR160	Serine carboxypeptidase; transcription factor; auxin-responsive factors (ARF10)	Up	[119, 120, 123, 125, 135–137]
miR162	Dicer-like proteins	Up	[138, 139]
miR164	NAC transcription factors	Up	[119, 135, 136, 140]
miR166	HD-ZIP transcription factors	Down	[135, 138, 141, 142]
miR166a-3p	Kinesin-1-like protein PSS1; transport		[129]
miR167	Auxin-responsive factors (ARF8)/cation/H+ exchanger	Up/down	[125, 126, 130, 136–140, 143]
miR167c-5p	SCF ubiquitin ligase complex		[129]
miR167f-3p_L-1R+1	Ubiquitin ligase complex; lysosomal alpha-mannosidase		[129]
miR168	ARGONAUTE1 (AGO1)	Up/down	[119, 125, 136, 138, 140]
miR168a-5p	WRKY; Cul4-RINGE3 ubiquitin ligase		[129]
miR168a-3p-L-3	Ubiquitin domain containing protein DSK2b		[129]
miR169	HAP2 transcription factors CAAT-binding factor/NFYA/MDIS1-interacting receptor-like kinase 1/protein high chlorophyll fluorescent 107	Up/down	[117, 119, 124–126, 130, 136, 137, 139, 141, 142, 144, 145]
miR169o	NF-YA1, NF-YA4, NF-YA10, NF-YA11, and TRA		[146]
miR169d-5p	Protein serine/threonine kinase; ABC transporter; C family member 13		[129]
miR169d-3p-L-2R+2	Galactinol-sucrose galactosyltransferase 2; E3 ubiquitin-protein ligase		[129]
miR169f-5p	NF-YA2		[119]
miR169(n, o)-5p	NF-YA4/NF-YA10		[119]
miR169r-5p	NF-YA1/NF-YA3		[119]
miR170	NSP2 transcription factor protein		[147]
miR171	Scarecrow-like transcription factors (SCL)/GAGA binding-like	Up/down	[121, 123, 125, 126, 130, 138]
miR171a	DELLA proteins RGL2; malate dehydrogenase		[129]
miR171a-3p	GRAS transcription factor/similar to scarecrow-like 6		[119]
miR171i-3p	GRAS transcription factor		[119]
miR171e-5p_2ss2GA21AG	Protein binding		[129]
miR172	AP2 like transcription factors	Up	[136–138]
miR172b-5p			[129]
miR172(a, d)-3p	AP2/EREBP family transcription factor		[119]
miR262	F-box/kelch-repeat protein	Down	[120]
miR319	TCP transcription factors/MYB transcription factor; Dnaj protein	Up/down	[125, 126, 129, 133, 136, 143]
miR319a-3p.2-3p_R+1	SCF ubiquitin ligase complex; MYB		[129]
miR319(a-3p.2,b)-3p	OsPCF6/TCP21		[119]
miR393(a, b)-5p	Auxin signaling F-box 2/OsTIR1		[119]
miR393-5p_L-1R+1	Auxin-activated signalling; protein transport inhibitor response 1; LRR kinase		[129]
miR393	Auxin receptors (TIR1/F-box AFB)/cyclin-like F-box	Up	[125, 126, 130, 133, 137, 138, 147]
miR394	F-box protein	Up/down	[139]
miR394-5p	SCF ubiquitin ligase complex; glutathione transferase activity		[129]
miR395	ATP sulfurylase; sulfate transporters (APS/AST)	Up/down	[120, 123, 125, 133, 136, 146]
miR396	Growth regulating factor (GRF)	Up/down	[121, 125, 138, 143, 148]
miR396a-5p	Phosphatidylinositol 4-phosphate 5-kinase 1; glutamate dehydrogenase; response to GA		[129]
miR396(a, b)-5p	OsGRF7		[119]
miR396c-5p	GTPase activity; HSP protein; RPM1		[129]
miR396e-5p	Transcription factor TFIID; ribonuclease P activity		[129]
miR396ef	s growth-regulating factors (OsGRFs)		[149]
miR397	Laccases, beta-6-tubulin/L-ascorbate oxidase/LAC17	Up/down	[120, 125, 127, 133, 136, 139, 147, 148]
miR398	COX5b-1; CCS1 COX (CSD)/IAA-amino acid hydrolase ILR1/GATA31	Up	[121, 123, 126, 136, 139, 147, 148, 150–153]
miR399	Ubiquitin conjugase E2/UBC24	Up/down	[123, 125, 129, 131, 136, 138, 143, 154, 155]
miR399a	Oxidoreductase; transcription factor		[129]
miR408	Plantacyanin laccases/superoxide dismutase [Cu–Zn]1A/SOD1A	Up/down	[120, 123, 125, 128, 131, 133, 136, 139, 147]
miR408_L-1	L-ascorbate oxidase; LPEAT1-like		[129]
miR408-p5_1ss20GA	Brassinosteroid insensitive 1		[129]
miR414	bZIP transcription factor	Down	[120]
miR419	Organic anion transporter		[147]
miR1425-5p	PPR domain-containing protein		[119]
miR444	MADS-box	Up	[119]
miR528	POD, SOD	Up	[136, 139, 140]
miR528-5p	Lectin-rich repeat receptor kinase		[129]
miR530-5p-R+1	Fructose-bisphosphate adolase; HSP70		[129]
miR530-5p_R+1_1ss20AG			[129]
miR530-5p-L+1	Fructose-bisphosphate aldolase; SCF ubiquitin ligase complex		[129]
miR531_L-4R+1-1ss5CT	NAD(P)H dehydrogenase; ABC transporter		[129]
miR780	Na^+^/H^+^ antiporter	Up	[123]
miR815c-p5_2ss6CT21CT	UDP-arabinose 4-epimerase 1-like; transcription factor TGA2-like; Dnaj		[129]
miR826	Alkenyl hydroxyalkyl producing 2	Up	[123]
miR827	Ubiquitin E3 ligase with RING and spx-domain-contain; phenylalanyl-tRNA synthetase; WPP domain-interacting protein 2; BTB/POZ domain-containing protein	Down	[123, 129, 136, 139, 147]
miR829	AP2 domain ethylene response	Up	[123, 133, 156]
miR839		Up	[123]
miR840	Basic helix-loop-helix (bHLH) DNA-binding protein		[147]
miR846	Jacalin lectin family protein	Up	[123, 156]
miR850		Down	[123]
miR854	UDP-glucuronic acid decarboxylase 2/ethylene-responsive transcription factor/GRAS transcription factor		[147]
miR857	Laccase	Down	[123]
miR858	Myb transcription factor		[130]
miR863	Transducin/WD40 repeat-like other RNA	Up	
miR1040	Acylsphingosine kinase/hypersensitive-induced response protein 1	Down	[120]
miR1074	LRR receptor-like serine/threonine-protein kinase		[147]
miR1088	CoA ligase-like 3		[147]
miR1127b-3p-1ss12TC	UDP-glucuronate decarboxylase; SCF ubiquitin ligase complex		[129]
miR1127b-p5-1ss3TC			[129]
miR1133	bZIP transcription factor superfamily protein		[147]
miR1137a-p3_1ss9CT			[129]
miR1137a-p5_2ss9GA20GC	DNA mismatch repair protein MSH6		[129]
miR1214	Peroxidase 2-like/F-box domain containing protein		[147]
miR2097	Translation initiation factor eIF-2B subunit delta		[147]
miR2199	MDIS1-interacting receptor-like kinase 1		[147]
miR2275-p5	LRR receptor-like serine/threonine-protein kinase FLS2; brassinosteroid insensitive 1/stress response gene TaPRP/signal transduction-associated genes TaWRK and TaSPK/trafficking genes TaAAT, TaNTA, and TaIM		[129, 157]
miR2630	Leucine-rich repeat receptor-like serine/threonine-protein kinase		[147]
miR2864	Sphingosine kinase 1		[147]
miR2916-p3-2ss2TC17CA	Fructose-bisphosphate aldolase; PRM1; peroxidase		[129]
miR2916-p5-2ss2AG17TG	Protein serine/threonine kinase; glutathione S-transferase (GSTF) 1; DELLA		[129]
miR2919	F-box protein		[147]
miR2948	LRR receptor-like serine/threonine-protein kinase	Down	[120]
miR3454	Protein kinase family protein		[147]
PC-5p-3645-2157	Metalloendopeptidase; SCF ubiquitin ligase		[129]
miR3706	Pentatricopeptide repeat-containing protein		[147]
miR3946	ABC transporter G family member 41/SBP-transcription factor 5		[147]
miR3979-3p	PPR domain-containing protein		[119]
miR4350	Cyclin-A2/putative ubiquitin-like-specific protease 1B		[147]
PC-3p-4780_1750	Aspartic endopeptidase; transcription factor		[129]
miR5059	Glycosyltransferase family 61 protein		[147]
miR5070	Transport inhibitor response 1 protein		[147]
miR5262	F-box/kelch-repeat protein	Down	[120]
miR5301	Harbinger transposase-derived nuclease	Down	[120]
miR5384-p5	UDP-glycosyltransferase 83A1-like; ethylene-responsive transcription factor 1B		[129]
miR5721	Auxilin-related protein 2	Down	[119]
miR6148	Hydroxycinnamoyl transferase	Down	[119]
miR6224	RNA-directed RNA polymerase	Down	[119]
miR6300-p5	Protein RPM1; response to nitrate		[129]
miR9652-5p	Retrograde transport		[129]
miR9653b-p3	Protein binding		[129]
miR9655-p5	Transcription factor; oxidation-reduction		[129]
miR9657a-3p	Serine-type endopeptidase inhibitor activity; amino acid transport; WRKY		[129]
miR9658-3p	SCF ubiquitin ligase complex; RPM1		[129]
miR9664–3p-L-1	RPM1 protein; zinc finger CCCH domain-containing glucosidase		[129]
miR9666a-3p	Alternative splicing regulator; regulator protein NPR5		[129]
miR9666b-3p	DNA-directed RNA polymerase II subunit I/stress response		[129]
miR9672a-3p-L+2R-2	Replication factor C subunit 1		[129]
miR9672b-p5	LRR receptor-like serine/threonine-protein kinase FLS2-like		[129]
miR9674b-5p	SCF ubiquitin ligase complex; calcium-dependent protein kinase 20-like		[129]
miR9772a-5p-L-2R+2	SCF ubiquitin ligase complex		[129]
miR9776-L-1R+1-1ss18AG	Cell division; O-methyltransferase		[129]
miR9779-p3	Hexose transmembrane transport		[129]
